# Editorial: Challenges in Inflammatory Bowel Disease: Current, Future and Unmet Needs

**DOI:** 10.3389/fmed.2022.979535

**Published:** 2022-07-18

**Authors:** Antonietta Gerarda Gravina, Raffaele Pellegrino, Fabiana Zingone

**Affiliations:** ^1^Hepatogastroenterology Unit, Department of Precision Medicine, University of Campania Luigi Vanvitelli, Naples, Italy; ^2^Gastroenterology Unit, Department of Surgery, Oncology, and Gastroenterology, University of Padova, Padua, Italy

**Keywords:** Inflammatory Bowel Disease, Crohn's Disease, Ulcerative Colitis, biologic therapy, COVID-19

## Introduction

Inflammatory Bowel Diseases (IBD), to which Crohn's Disease (CD) and Ulcerative Colitis (UC) belong, are chronic digestive disorders with a natural history of remitting-recurrent course that can directly impact the patient's quality of life. UC is limited to the colon and rectum with continuous and non-transmural involvement while CD has a much more complex disease topography as it can affect the entire gastrointestinal tract from the mouth to the anus with discontinuous and transmural involvement. IBD are associated with numerous extra-intestinal manifestations (such as articular, ocular, cutaneous, urogenital, and cardiorespiratory) that greatly complicate the basic clinical picture resulting in a disease phenotype that can be extremely varied. Treatment relies on pharmacological, nutritional even surgical interventions. Often the patient, in case of intolerance or failure of conventional therapy or in case of dependence or refractoriness to steroid therapy, undergoes immunosuppressive therapy also with biologic drugs. The latter have positively revolutionized the prognosis and course of IBD. However, IBD patients on immunosuppressive therapy are exposed to a wide range of infectious agents for which appropriate preventive measures are recommended, even considering the incipient Severe Acute Respiratory Syndrome CoronaVirus 2 (SARS-CoV-2) pandemic. The purpose of this Research Topic was to gather new evidence on the complex needs of patients with IBD.

## Current and future IBD needs

Having a non-single-perspective view of the IBD patients but looking at them from a multidimensional perspective contemplating intra-individual, inter-individual, and inter-national differences, is certainly merit that research must constantly pursue. Some of the papers on our Research Topic have helped in this regard.

Elbadry et al., for example, studied the clinic-epidemiological characteristics of Egyptian IBD patients in a large sample of 1,104 patients, observing, among various findings, a greater presence of male patients. Always staying within the gender characteristics Shehab et al. found, in a retrospective multicenter study of more than one thousand patients, that in IBD patients on therapy with Infliximab, anti-drug antibody levels were higher in males than in females. Confirming the finding, trough levels were also lower in the male sex. The same finding was not confirmed for adalimumab.

Among the needs of IBD patients are those undergoing stoma placement, who are adapting to a new routine and new disease management with no small psychological repercussions, which have to be of extreme interest. Wang et al. observed in a stoma court of IBD patients how there is a moderate degree of perceived stigma with a low-moderate level of self-efficacy that increased in patients with higher educational status. Having a close person who accepts the stoma helps the patient in relative self-awareness and self-acceptance.

Special attention should be also paid to patients with CD at risk of common complications, including stenosis and intestinal obstruction. Xia et al. presented two clinical cases, of which in one there was endoscopic capsule retention in the small bowel of a patient with CD with concomitant bowel obstruction and, in the second case of a patient with a CD with rare intestinal obstruction from mesangial hernia and ileal stenosis. Both cases were treated with minimally invasive surgery. Thus, the authors emphasized careful evaluation of bowel patency by the clinician before performing an endoscopic video capsule.

Nardone et al. provided us with a detailed review of the role of intestinal ultrasound in the clinical management of IBD, also providing insights into the detection of post-surgical recurrence in CD. Tien et al. conducted, moreover, a retrospective study investigating the impact of IBD and specific IBD therapy on the risk of developing hyperlipidemia, comparing more than 10,000 patients with IBD with a control group. Within the IBD group, the subgroup not receiving any therapy presented an increased risk compared with patients being treated for IBD. IBD therapy appeared to downregulate the expression of hepatic lipogenic genes and, therefore, the authors stigmatized screening for hyperlipidemia in the management of IBD. Certainly, the study of non-invasive markers to monitor and assess the severity of IBD is important for our clinical practice. Lin et al. found, by examining different indices, how some of these including the C-reactive protein-to-lymphocyte ratio and C-reactive protein-to-albumin ratio can help in this field.

Khoramjoo et al., in addition, in an interesting mini-review examined the role of three proliferative pathways namely Wnt, Notch, and Hippo in IBD. This assumption draws its origin in the impairment and dysregulation of these pathways in IBD. Indeed, it emerged how these pathways are essential in maintaining an adequate bowel epithelial barrier that is impaired in inflammatory digestive diseases such as IBD. Modern techniques, such as Single Cell RNA Sequencing, have added knowledge to IBD pathogenesis. Serigado et al. have reviewed the role of this technique and found how it can provide remarkable potential in characterizing the different cytologic subpopulations involved in the pathogenesis of UC (such as BEST4+ cells, colonic M cells).

## The problem of the SARS-CoV-2 pandemic

Coronavirus pandemic disease 2019 (COVID-19) has inevitably resulted in treatment delays in IBD patients, especially those on biologic agent therapies. Li et al. provided a retrospective study that examined the risk factors for delaying treatment with Infliximab in patients with CD, with a propensity-matching score, to compare the effects of the same delay on short- and long-term outcomes. They identified a treatment delay rate, in a retrospective cohort of 53 patients, of 71.7% in Xiangya Hospital, China. They found that the CD Activity Index decreased less, over the course of the study time, in the delayed-treatment patient group with a higher long-term hospital readmission rate increased by 33%.

The safety of patients with IBD, especially on immunosuppressive therapy toward infectious risk is relevant, particularly considering the current SARS-CoV-2 pandemic status.

Shehab et al. studied the safety of the vaccine by examining short- (within 3 weeks of vaccine administration) and long-term (within 24 weeks) adverse events in a sample of 408 subjects (50% allocated in the IBD group and 50% in a sample of healthy controls). A good safety profile and absence of serious adverse events emerged with some local adverse events (pain at the injection site observed in patients with IBD after the first dose and fatigue after the second dose). An additional possibility is, as described in previous evidence, that patients on immunomodulating therapies, may present an attenuated antibody responses after anti-SARS-CoV-2 vaccination. Again Shehab et al. studied, in 162 patients on combination therapy (with azathioprine) with Infliximab, the immunogenicity of the vaccine and observed how the levels of specific anti-SARS-CoV-2 IgG as well as neutralizing Ig were significantly higher in recipients of a third booster dose than in those vaccinated with only two doses. They also observed how the booster dose increased the rate of patients who developed positive anti-SARS-CoV-2 IgG antibodies (96.5 vs. 90%) and neutralizing antibodies (100 vs. 88.9%).

## Author contributions

AG and FZ contributed equally to the editorial process of this Research Topic and the drafting of this editorial. AG, RP, and FZ equally contributed to the writing of this manuscript. All authors contributed to the article and approved the submitted version.

## Conflict of interest

The authors declare that the research was conducted in the absence of any commercial or financial relationships that could be construed as a potential conflict of interest.

## Publisher's note

All claims expressed in this article are solely those of the authors and do not necessarily represent those of their affiliated organizations, or those of the publisher, the editors and the reviewers. Any product that may be evaluated in this article, or claim that may be made by its manufacturer, is not guaranteed or endorsed by the publisher.

